# Preventing and Protecting Against Internet Research Fraud in Anonymous Web-Based Research: Protocol for the Development and Implementation of an Anonymous Web-Based Data Integrity Plan

**DOI:** 10.2196/38550

**Published:** 2022-09-12

**Authors:** Kris L Hohn, April A Braswell, James M DeVita

**Affiliations:** 1 College of Health and Human Services School of Social Work University of North Carolina Wilmington Wilmington, NC United States; 2 College of Health and Human Services School of Nursing University of North Carolina Wilmington Wilmington, NC United States; 3 Watson College of Education University of North Carolina Wilmington Wilmington, NC United States

**Keywords:** data integrity protocol, DIP, anonymous online research, research fraud, IP geolocation, steps and procedures, pre-post intervention outcomes, online, research, protocol, data, privacy, participation, validity, reliability, preliminary, analysis

## Abstract

**Background:**

Data integrity is a priority in any internet research study; it should be maintained to protect the safety and privacy of human participants and to maintain the validity and reliability of research findings. However, one noteworthy risk of web-based research is fraudulent respondent activity. When investigators must utilize anonymous web-based recruitment techniques to reach hidden and expanded populations, steps should be taken to safeguard the integrity of data collected.

**Objective:**

The purpose of this paper is to present a novel protocol in the form of an anonymous web-based research data integrity plan (DIP) protocol that outlines steps for securing data integrity while conducting anonymous web-based data collection.

**Methods:**

In this paper, we discuss a protocol regarding the development and implementation of a specific DIP in response to fraudulent activity in an original large-scale mixed methods study launched in April 2021. Four primary steps, each with a set of affiliated procedures, are presented: (1) defining the risks, (2) planning research protocols, (3) securing data collection and recruitment, and (4) determining enrollment.

**Results:**

Following the relaunch of a large-scale original study and implementation of the DIP protocol, preliminary analyses demonstrated no fraudulent activity. A pre-post analysis is underway to evaluate the effectiveness of the DIP strategies from February 2022 through May 2023.

**Conclusions:**

Implementing the DIP protocol could save valuable research time, provides a process to examine data critically, and enables the contribution of rigorous findings to various health fields.

**International Registered Report Identifier (IRRID):**

DERR1-10.2196/38550

## Introduction

### Background

Researchers cannot minimize the importance of data integrity when conducting research. Data integrity is connected to both excellence and quality in research and science for policy [1]. Data are the currency of the digital world and have become necessary for many disciplines to carry out day-to-day activities [2]. The use of technology and electronics worldwide has introduced an array of innovative and instrumental possibilities for scientists and researchers [3]. However, these possibilities have emerging risks. Data integrity risks can directly or indirectly interrupt the recruitment, data collection, data analysis, or interpretation phase of research and ultimately threaten the integrity of outcomes and research findings; if not identified and eliminated, such risks could lead to inappropriate and potential harmful recommendations for practice as well. The threat of hackers or fraudulent data interrupters is most concerning because “altering the grounds of data truth has the potential to destroy prominence (both personally and professionally) and allows intruders to intertwine with cyber security, public health, and safety” (p 854) [2]. The aim of this paper is to introduce an anonymous web-based research data integrity plan (DIP) focused on preventing and protecting against internet research fraud. Moreover, we developed procedures after identifying a data integrity threat in the recruitment phase of a web-based mixed methods research study.

### Background on Fraudulent Research Activity

The terms “phishing,” farming,” and “hacking” are not uncommon concepts in the world of internet technology. However, scientists do not frequently consider fraud in developing survey research. Internet research fraud is becoming a growing concern. Fraudulent users are (1) eligible persons who take a research survey more than once without wrongful intent, (2) eligible persons who repeat a research survey for additional compensation, or (3) ineligible persons who participate in a research survey once or more to benefit from compensation [4].

The use of web-based survey tools and distribution methods (eg, social media) for research can be beneficial for reaching a diverse participant pool but problematic for ensuring data quality. Web-based surveys may be instrumental in reaching stigmatized populations (eg. men who have sex with men) or in seeking information on stigmatized topics (eg, sexual health and drug use) [5-7]; however, there is potential for fraud that could compromise the validity and reliability of data collected from these methods [5-9].

One potential fraud can occur through inattentive responses, which Maniaci and Rogge [9] found to be an issue in their research. While inattentive responses can negatively affect the results of a study, they can also be measured and addressed as part of fraud monitoring [9]. Similarly, attempts at “phishing” can be monitored and managed by researchers as well. In one study, Pozzar et al [7] found that a survey distributed via social media platforms included 100% of fraudulent responses from among the initial ≥270 responses. While troubling, the authors were able to detect the issue, adapt their distribution and screening approaches, and recommend others develop a protocol for monitoring fraud in their research [7]. Ballard et al [5] also emphasized the development of a protocol. They described 3 components that help minimize fraud: “Researchers should have a fraud detection algorithm in place before data collection to ensure that (1) data needed for fraud detection are being collected; (2) the informed consent document can describe that surveys will be evaluated for fraud and what the consequences are for incentives, and (3) fraud can be monitored in real time” (p 9).

Indeed, multiple authors described the data they collected and used to monitor fraud, including geolocation, physical address, email ID, and phone number, among others. For example, Pozzar et al [7] discussed matching the time when a user completed a survey to geolocation data—one of the multiple authors to suggest that geolocation data are best used in connection with other data but not as fraud check alone [5,6]. Bowen et al [6] described using repetitive patterns in usernames, passwords, and email IDs submitted by respondents to register and access incentives because “the promise of even $15 may increase the rate of spurious submissions” (p 9). The requirement of physical mailing addresses for incentives to be sent rather than automatically via email is one way to monitor fraud [5]. Researchers can use physical addresses to match geolocation data. Another potential deterrent is requiring participants to create a unique ID (credential) and log-in information to participate and retrieve incentives [6,10]. Thus, there are many ways in which researchers can help minimize the adverse effects of fraudulent responses.

### Defining Geolocation and Geolocation Problems

One way to review and investigate research fraud is through safe and ethically approved use of geolocation. An IP address is one of many components that aid in geolocation; nonetheless, there are numerous issues with how accurate geolocation is [5-7,11]. An IP address, more specifically IP version 4 (IPv4), is a 32-bit address structure that serves 2 primary functions: addressing (the set of rules for networks and hosts to follow to ensure that messages move across the internet efficiently) and fragmentation (essentially breaking down a message into smaller bits of information to transfer across the internet and then putting the data back together in the correct order at its destination) [11]. The next evolution of IP addresses, version 6 (IPv6), accounts for the high demand for IP addresses owing to the ever-increasing use of the internet and the normalization of individuals having multiple devices that connect to the internet [11]. While IPv4 is of 32 bits, IPv6 has 128 bits, allowing for 340 undecillion addresses (or “340 trillion trillion trillion addresses”), creating more than adequate room for continued expansion (p 18) [11].

It is essential to understand the limitations of IP address geolocation [12-14]. First, the accuracy of IP address geolocation varies between IPs. Owing to early adoption and less precise *touchstones* (“reliable network landmarks”), IPv6 is currently less accurate than IPv4 [15,16]. Research innovators who address the network limitations are proposing ways to build the IPv6 network off the IPv4 *touchstones* [16]. Second, country- versus city-level accuracy can vary greatly [13-15]. Many geolocation databases report 60%-99.99% accuracy at the country level and only 30%-80% accuracy at the city level [17]. Third, the type of network the user is communicating from may impact IP accuracy, with mobile systems reporting far less precision than broadband [14,15,18]. Fourth, and perhaps most importantly, virtual private networks (VPNs) complicate geolocation abilities. A VPN routes a user’s IP address through a private network so that the user’s internet traffic is encrypted [19]. A user could choose to route their IP through another state and another country entirely.

## Methods

### Methods Overview

The eligibility survey investigated in this study was reported in accordance with Eysenbach’s [20] Checklist for Reporting Results of Internet E-Surveys (CHERRIES) shown in Multimedia Appendix 1 (the CHERRIES Checklist [20] applied to anonymous web-based survey eligibility survey). The preintervention phase with the survey was carried out from April to September 2021 (during original study recruitment), while the postintervention phase was launched in February 2022 (initial study relaunch after DIP initiation) through May 2023.

During the recruitment phase of our study, the research team recognized patterns and suspicious activity that led to the identification of research fraud and data integrity risk. The research methods (anonymous web-based recruitment) and protection of the target population (sexual and gender minority adolescents) made the research more permeable to fraudulent activity. The use of participant compensation for time and web-based anonymous data collection via an electronic survey are common in quantitative, qualitative, and mixed methods research. However, Teitcher et al [4] reported that these elements show an increased potential for Internet research fraud. In the process of (1) integrating specific guidelines for special populations from the Department of Health and Human Services, (2) upholding human subject protection standards for minors outlined in the for the Protection of Human Subjects of Biomedical and Behavioral Research’s Belmont Report [21], and (3) offering compensation for study participation, we increased the risk of a data integrity breach. When we determined that research fraud was present and data integrity was at risk, the study was paused to seek institutional review board approval for a newly developed internet research fraud prevention and protection protocol.

The DIP protocol’s steps and procedures are outlined in Multimedia Appendix 2 [22-28]. Of note, when engaging with research with web-based recruitment, researchers should be aware of the inherent risks of fraudulent activity. Studies conducted on the internet are not without risk to human subjects. Therefore, researchers should examine and apply principles of *respect for persons*, *beneficence,* and *justice* outlined in the National Commissions for the Protection of Human Subjects of Biomedical and Behavioral Research’s Belmont Report [21]. Moreover, the Office for Human Research Protections in the US Department of Health and Human Services outlines special considerations for vulnerable research populations (ie, human fetuses, neonates, pregnant women, children, and prisoners) [29]. These populations require individual assessment for risk and protection, even for internet recruitment and enrollment procedures.

### Ethics Approval

This study was approved by the University of North Carolina Wilmington’s institutional review board (#20-0126).

## Results

As of this writing, a pre-post analysis is in the data collection phase to assess the effectiveness of the DIP strategies outlined in Multimedia Appendix 2 from February 2022 through May 2023. From April 2021 to August 2021, we enrolled 12 participants for semistructured qualitative interviews for a mixed methods study. Before launching the DIP, various indications of fraudulent activity were noted. These include the following: (1) several surveys were entered by respondents but not completed, (2) a rush of survey time stamps was found in the same 1-15–minute period, and (3) exact or similar respondent locations were found among many respondents. The team employed DIP steps 1-3 to secure the survey and then utilized step 4 to review existing data. Researchers determined that 45 unenrolled survey respondents and 3 enrolled respondents were ineligible upon critical analysis of the survey respondent data. Preliminary examination has revealed zero instances of fraudulent activity in the survey from our original study after implementing the DIP in February 2022.

## Discussion

The researchers anticipated that implementing the DIP would decrease fraudulent activity in the eligibility survey. This hypothesis remains supported.

### Strengths and Limitations

One strength of this study includes the integration of interdisciplinary evidence supporting the development of the DIP protocol. The evidence-based protocol provides future researchers with specific guidance on how to protect their data integrity. When working with vulnerable populations where anonymity is critical, this protocol will enable teams to secure privacy while not jeopardizing the data collection. One limitation is the inability of researchers to control or reasonably estimate the number of participants who may have viewed the recruitment text on the various platforms and not entered the landing page [30,31]. This is one of the inevitable limitations of web-based convenience sampling. To counter this limitation, researchers strategically asked participants who were enrolled and consented to an interview to identify which platform they first learned about the study. This will enable researchers to identify which platforms were most successful for recruitment in future studies.

### Future Directions

#### Overview

Our research team quickly adapted following the experience of research fraud. In addition to the DIP protocol, we offer the following learnings to aid researchers in streamlining efforts for timely, successful, rigorous, and protected data collection in future studies.

#### Overcoming Barriers

Research teams may feel defeated when identifying fraudulent attempts to join a study. This deception can be particularly confusing and frustrating when inclusion criteria involve vulnerable populations [5]. Research teams will be optimally prepared if they understand fraud risk in advance, employ methods to uncover these risks, and embrace empathy, even for perpetrators of the fraud. This allows for a quicker team recovery. Harboring feelings of anger and resentment may slow the positive progress of the team’s mission. The method of reframing, also referred to as *cognitive restructuring*, is a simple technique to transition from confusion to understanding and empathy [32].

#### Timely Review

A critical strategy for web-based research recruitment is constant review of incoming data [15,16,27] and smooth communication. Delegation of a DIP protocol is essential for careful review and prevention of research burnout. Research teams should identify who oversees reviewing IP and location issues, how to share that information with the team, who will enact the screening procedures, and how to respond to ineligible participants, owing to the time required to complete fraud prevention strategies [33]. The research team should also consider the benefit of forming a co–principal investigators’ (>1 principal investigator) structure to help balance efforts of fraud prevention protocols.

### Implications and Conclusions

Medical, health, and other applied science disciplines demand rigorous internet research methods to produce valid and reliable findings. When the results of research are threatened by internet fraud, research budgets are impacted, study timelines are negatively affected, and data lack quality [4]. One essential precursor to conducting rigorous internet medical research is a prevention protocol. Research data integrity involves more than just having a correct data set. Preserving the integrity of research data has critical implications for organizational policy, future development related to health informatics, and the future of internet medical research methods.

## Appendix

Multimedia Appendix 1 CHERRIES Checklist [20] Applied to Anonymous Web-Based Eligibility Survey.

Multimedia Appendix 2 DIP Protocol's Steps and Procedures to Prevent Web-Based Research Fraud. DIP: anonymous web-based research data integrity protocol.

## Abbreviations

CHERRIES: Checklist for Reporting Results of Internet E-Surveys
DIP: anonymous web-based research data integrity protocol
IPv4: IP version 4
IPv6: IP version 6
VPN: virtual private network

## Data Availability

The data sets generated in this study are not publicly available owing to the sensitive nature of the original study and to ensure the protection of the vulnerable population studied.
