# Supplementary material for: Preventing and Protecting Against Internet Research Fraud in Anonymous Web-Based Research: Protocol for the Development and Implementation of an Anonymous Web-Based Data Integrity Plan
Source: JMIR Res Protoc. 2022 Sep 12;11(9):e38550. doi: 10.2196/38550 (PMC9513686; doi:10.2196/38550)
Supplement: Multimedia Appendix 1 [file resprot_v11i9e38550_app1.pdf]

Multimedia Appendix 1: CHERRIES Checklist[20] Applied to Anonymous Web-Based Eligibility Survey

Item Category	*Checklist Item*	*Explanation*
**Design**		
	Describe survey design	Target Population: sexual and gender minorities, ages 11-19, located in the United States.
**IRB (Institutional Review Board) approval and informed consent process**		
	IRB approval	Approved by the University of North Carolina Institutional Review Board #20-0126
	Informed consent	Interested participants found text, audio, and video information about the study purpose and informed consent on the recruitment website and recruitment survey. The investigators created a video with all involved to describe all aspects of the study purpose, data privacy, anonymous data collection, and incentives.
	Data protection	No personally identifiable information was collected from participants.
**Development and pre-testing**		
	Development and testing	State how the survey was developed, including whether the usability and technical functionality of the electronic questionnaire had been tested before fielding the questionnaire.
**Recruitment process and description of the sample having access to the questionnaire**		
	Open survey versus closed survey	Open Survey
	Contact mode	Contact was only made to the participants upon successful completion of recruitment qualification survey. Interested participants had to click on the recruitment landing page link from the study announcement, access the survey, and complete the survey online before investigators contacted them via provided phone number.

Item Category	*Checklist Item*	*Explanation*
	Advertising the survey	Participants were able to find or receive recruitment information from social media sites (Reddit and Facebook) as well as age-appropriate email listservs. The study was described in detail on the recruitment landing page that interested participants could link to from the recruitment call.
**Survey administration**		
	Web/E-mail	The e-survey was linked in the recruitment landing page. If interested participants wanted to complete the survey, they were routed to the Qualtrics platform that hosted the survey. Qualtrics was programed to automatically capture and save their responses.
	Context	The recruitment text was sent out to sexual and gender minority specific sub-Reddits and Facebook groups, listservs, and printed out for display in several local sexual and gender minority accepting healthcare locations in Southeast North Carolina.
	Mandatory/voluntary	The survey was voluntary and anonymous.
	Incentives	Upon completion of an online interview with the research team, participants received a $15 online gift card incentive. No incentive was provided for those who completed the eligibility survey.
	Time/Date	April 2021-on going
	Randomization of items or questionnaires	Survey items were not randomized due to the nature of the survey (to establish eligibility to be involved with the research interview)
	Adaptive questioning	Adaptive questions were utilized based on responses to age, location, willingness to engage in interview, contact information and consent/assent. Should interested participants be found ineligible (such as out of the age range or location) then a thank you message and statement of eligibility was provided.
	Number of Items	Up to 14 questions were displayed depending on responses.
	Number of screens (pages)	Up to 13 pages were utilized depending on responses.

Item Category	*Checklist Item*	*Explanation*
	Completeness check	Mandatory questions were included for participants to move forward from page to page.
	Review step	Respondents were able to utilize the back button on any question to review their responses.
**Response rates**		
	Unique site visitor	After implementing the DIP plan, Qualtrics was set to block repeat IP addresses.
	View rate (Ratio of unique survey visitors/unique site visitors)	View rates were unavailable due to the mandatory response questions. Participants were not able to view all questions without first responding.
	Participation rate (Ratio of unique visitors who agreed to participate/unique first survey page visitors)	The recruitment rate is not yet available as data collection is till ongoing (April 2021-ongoing) (present day, July 2022)
	Completion rate (Ratio of users who finished the survey/users who agreed to participate)	Completion rate not yet available.
**Preventing multiple entries from the same individual**		
	Cookies used	Qualtrics identified repeat IP addresses and blocked them from more than one entry into the eligibility survey.
	IP check	IP addresses were checked.
	Log file analysis	Prior to DIP plan, data was downloaded by the researchers and manually searched for repeat users. The first entry of any user was utilized, and duplicates were removed.
	Registration	Registration was not utilized.
**Analysis**		
	Handling of incomplete questionnaires	No incomplete surveys were utilized due to mandatory responses and the purpose of the eligibility survey (to identify eligible interested participants for a qualitative interview).
	Questionnaires submitted with an atypical timestamp	Timestamps were collected but only utilized as supportive evidence of fraudulent activity.
	Statistical correction	No statistical corrections needed or utilized.
