# Supplementary material for: Preventing and Protecting Against Internet Research Fraud in Anonymous Web-Based Research: Protocol for the Development and Implementation of an Anonymous Web-Based Data Integrity Plan
Source: JMIR Res Protoc. 2022 Sep 12;11(9):e38550. doi: 10.2196/38550 (PMC9513686; doi:10.2196/38550)
Supplement: Multimedia Appendix 2 [file resprot_v11i9e38550_app2.pdf]

Multimedia Appendix 2: DIP Protocol’s Steps and Procedures to Prevent Web-Based Research Fraud

Step	*Procedure*	*Explanation*
**Step 1: Defining the Risks**		
	Procedure A: Determining Risks of Online Recruitment	Researchers must investigate the risk and benefits of recruiting research participants online and cross examine those risks with the target population (i.e. vulnerable or not).[10,23]
	Procedure B: Adapting to Risks of Anonymity	Researchers should conclude if they must recruit “hidden” and/or vulnerable communities and build anonymity into recruitment and data collection if warranted. This step increases the protection of personally identifiable participant data.[3,5]
	Procedure C: Balancing Risks of Advertising Research Compensation	Researchers should refrain from advertising research compensation in recruitment calls, email blasts, and other methods of digital recruitment text.[4,6] Researchers should locate this information in the consent/assent text after pre-screening.
**Step 2: Planning Research Protocols**		
	Procedure A: Creating Recruitment Landing Page.	Researchers should develop a simple, shareable webpage as a recruitment landing site. This could include an original website or use of a learning management system page. This should include the project title, name, and contact information for lead researchers, a written description of the project, a video of the research team discussing the project, and a link to the eligibility screening survey. Researchers should embed a link to the recruitment landing page in recruitment advertisements.
	Procedure B: Developing Eligibility Assessment Plan	Researchers should collectively establish the parameters for inclusion criteria in the study. Researchers should translate parameters into a screening survey to assist the team in recruiting eligible participants. Specific steps for evaluating eligibility should be written in a simple plan for all research team members. Research tools can collect IP addresses as a form of geolocation to provide additional confirmation about eligible participants. Many survey platforms automatically collect IP addresses from survey responders, and it is the responsibility of the researcher to turn this setting off.[24,25] While IP addresses do not identify a person’s name, physical address, or other personally identifiable attributes,[26] is considered privileged information. Collecting this data should be approved by the researcher’s IRB board before launching a survey.

Step	*Procedure*	*Explanation*
	Procedure C: Establishing a Communication Plan	Researchers should employ additional follow-up strategies before engaging the participant in the study. If all communication occurs anonymously online, some direct contact form will enable the researchers to sort out fraudulent users. Phone numbers are less identifiable than an email address that may contain a participant's name or other identifiable information. Creating a private research phone line with a paid or free national online service recommended for interested participants so researcher staff can keep personal phone numbers private.[27]
**Step 3: Securing Data Collection and Recruitment**		
	Procedure A: Creating an Original Screening Survey	Non-identifiable participant information should be collected that directly relates to the study eligibility criteria. Potential participants are directed to a survey screening prior to enrollment. Researchers should use this data as a verification tool in the final step. Researchers should identify all security features available in their survey platforms and select components that fit the parameters of their study. Researchers can use functions to prevent multiple survey submissions and verify respondents are not "bots" through a service such as reCAPTCHA. Survey platforms can also block specific search engines from including research surveys in search results.[25]
	Procedure C: Adding Screening Questions in Original Research Surveys	When screening questions are developed, specific validation steps can be embedded into the survey to improve the quality of collected data. The first step is adding requirements for questions to be answered (i.e., consent/assent, contact information, etc.) before moving forward. Prompts are then sent to notify the potential participant that an error was made in the survey.[24] Another feature offered by some survey platforms is adding a setting to verify that phone numbers collected from participants (if warranted) are valid US numbers. Survey users are notified if the phone number entered is not a valid-US phone number.[24]

Step	Procedure	Explanation
	Procedure D: Developing Unique Participant ID	Along with the foundational information collected in an eligibility screening, researchers should design a unique system for participants to create a research ID that will not be difficult to recall. This process allows researchers to uphold anonymous research data collection procedures and have a private and personalized pseudonym for every participant enrolled. An original ID is a system that uses numbers and letters from phone numbers, birthdays, and first and last names or other information a participant would not forget and would be able to replicate easily if asked again. Here is an example of one prompt (used in the original research discussed in this paper) which includes a 6-digit unique identifier: To best protect your identity, we make every effort not to collect names. Instead, please give us a personal ID we can use instead of your name. Create your 6-digit person ID using the following code: To best protect your identity, we make every effort not to collect names. Instead, please give us a personal ID we can use instead of your name. Create your 6-digit person ID using the following code:<br>*First letter of your birth month<br>*Last letter of your first name<br>*Last letter of your birth month<br>*First letter of your last name<br>*Fourth number of your phone number<br>*Last number of your phone number<br><br>As described in Procedure C above, adding a validation requirement to the unique identifier insists that a respondent complete the field before moving further through the survey.[24] Developing an ID that incorporates numbers and letters allows research team members to identify errors quickly.
**Step 4. Determining Enrollment**		
	Procedure A: Screening IP address	Researchers may save time by verifying that their surveys are programmed before launch to catch repeat IP addresses. However, it is essential to heed the advice of frequent critical review and assessment of any IP or geolocation data.[28] Should research teams discover their survey did not block repeat-IP addresses, a critical examination of the data may be needed. Research teams should sort and label problematic cases. Odd identical or practically identical IP addresses found can be identified in Excel using a simple color-coding technique (e.g. green=no problem identified; yellow=unlikely to be problematic closer review advised; orange=likely problematic with closer review needed; red=clearly problematic).

Step	*Procedure*	*Explanation*
	Procedure B: Screening Location	Researchers with country, region, state, or local inclusion criteria should consider location screening. Fraudulent activity can be captured by review of respondent location data. When browser-based survey platform records participant locations, this is captured in the form of approximated latitudinal and longitudinal coordinates.[29] Without the presence of a VPN and other methods to mask internet activity, these coordinates should not land outside of the general area of your research criteria. The research team may apply the color-coding scheme across cases.
	Procedure C: Screening Unique Participant ID.	Researchers should review unique participant ID's created and submitted by respondents. Research staff may be able to identify fraudulent activity when the respondent-created IDs vary significantly from the instructions alongside (e.g., all numbers are provided when a variety of numbers and letters are asked for). If mistakes occur alongside other areas of concern (IP address and location identified in Procedure A and B), the research team can identify this as a respondent that requires closer review before enrollment.
	Procedure D: Conducting and Assessing Brief Text Message Screening	If a participant is willing to share contact information with the research team, researchers can quickly conduct a brief text-message screening to verify eligibility further. The research team can reconfirm participant age, country, and select inclusion criteria. Research staff should be alerted to possible fraud when participants responses vary greatly from the data collected from original online survey responses reported.
	Procedure E: Conducting and Assessing Brief Phone Screening	Following the brief text screening, a research team member can set up a phone screening to confirm that a computerized or recorded voice is not heard or that the respondent is not a "bot." This initiates another level of eligibility screening by verbally confirming the screening questions. Phone call eligibility screening should consist of the same questions in the text message screening and will alert the research team member if a robot or computerized voice is detected.
	Procedure F: Finalizing Enrollment	After gathering the screening survey, IP address, and location data and conducting brief text and phone screenings, the researchers have implemented a protocol that contributes to decreased fraud risk. If the researcher is confident of eligibility during the phone screening, steps can be taken during the phone call to enroll the individual in the study officially or to discuss the next steps in the enrollment process— If participants are deemed ineligible by the research staff, a message outlining eligibility criterion should be sent via text or provided verbally over the phone to the respondent.
